# Functional Heterogeneity of Reelin in the Oral Squamous Cell Carcinoma Microenvironment

**DOI:** 10.3389/fonc.2021.692390

**Published:** 2021-08-17

**Authors:** Xinwen Zhang, Yong Fu, Zhuang Ding, Nisha Zhu, Mengxiang Zhao, Yuxian Song, Xiaofeng Huang, Sheng Chen, Yan Yang, Caihong Zhang, Qingang Hu, Yanhong Ni, Liang Ding

**Affiliations:** ^1^Central Laboratory of Stomatology, Nanjing Stomatological Hospital, Medical School of Nanjing University, Nanjing, China; ^2^Department of Oral Pathology, Nanjing Stomatological Hospital, Medical School of Nanjing University, Nanjing, China; ^3^Research Institute of Superconductor Electronics, School of Electronic Science and Engineering, Nanjing University, Nanjing, China

**Keywords:** Reelin, oral squamous cell carcinoma, cancer-associated fibroblasts, prognosis, lymphocyte subsets

## Abstract

**Background:**

Reelin, an extracellular glycoprotein, is expressed on neuronal cells and participates in neuronal migration during brain development. Recently, Reelin also has a vital role in carcinogenesis. However, its role in oral squamous cell carcinoma (OSCC) remains to be explored. The purpose of this study was to explore the roles of Reelin in OSCC.

**Methods:**

The expression of Reelin in cancer**-**associated fibroblasts (Reelin^CAF^) and tumor cells (Reelin^TC^) was analyzed by the Gene Expression Omnibus (GEO) database. Immunohistochemistry (IHC) was used to detect the spatial pattern of Reelin in 75 OSCCs. The diagnostic and prognostic values of Reelin were evaluated and also verified by The Cancer Genome Atlas (TCGA) database. Primary CAFs from 13 OSCC patients were isolated to confirm Reelin expression. Thirty-nine OSCC peripheral blood samples were used to analyze the change of immunocytes based on Reelin levels by flow cytometry. The relationship between Reelin and tumor immune microenvironment in head and neck squamous cell carcinoma (HNSCC) tissues was determined by TISIDB and the Tumor Immune Estimation Resource (TIMER) database.

**Results:**

In breast cancer, pancreatic cancer and rectal cancer, Reelin in CAFs was significantly upregulated compared with Reelin in TCs. The IHC results in OSCC also showed that Reelin levels were higher in CAFs. Upregulated Reelin^TC^ was related to a decreased pN stage and distant metastasis. Strikingly, patients with enhanced Reelin^CAF^ had a high risk of lymph node metastasis, poor worst pattern of invasion (WPOI), and distant metastasis, but showed comparable Ki-67 level in all OSCC patients, resulting in shorter overall survival (OS) and disease-specific survival (DSS). Unexpectedly, Reelin in tumor-infiltrating lymphocytes (Reelin^TIL^) was correlated with postoperative relapse. Patients with high Reelin^TIL^, but not Reelin^TC^ and Reelin^CAF^, had poor cytotoxicity of CD8^+^ T cells and higher ratio of CD4/CD8 in peripheral blood. However, Reelin was positively associated with tissue-resident B cells and NK cells in the tumor microenvironment.

**Conclusion:**

Reelin has a versatile function in distinct cell types during the development of OSCC *via* governing tumor cell and stroma microenvironment.

## Introduction

Head and neck squamous cell carcinoma (HNSCC) was the sixth most common cancer worldwide in 2018. Oral squamous cell carcinoma (OSCC) is the most common malignant tumor of the head and neck with a high mortality rate (1–3). Therefore, the potential predictors of OSCC are awaited to identify for guiding diagnosis and treatment. Reelin is a secreted extracellular matrix glycoprotein that is formed with 3,461 amino acids. It is secreted by Cajal-Retzius cells in the marginal zone and encoded by the RELN gene, which is located on chromosome 7q22. The molecular weight of Reelin is about 400 kDa (4–6). To date, a majority of Reelin-related studies have focused on neurological diseases, such as schizophrenia, autism, and Alzheimer’s disease because Reelin helps modulate the processes of neuronal migration and positioning during brain development (7–10). Importantly, Reelin is expressed in many extraneuronal tissues, including plasma, blood cells, liver, and intestine. However, the roles of peripheral Reelin remain unknown, especially in oral tissues with a large number of nerves (4).

Notably, alterations in Reelin expression are observed in cancers of nonneuronal origin (11). Specifically, the expression of Reelin is reduced in hepatocellular carcinoma, gastric carcinoma, colorectal cancer, glioblastoma, breast cancer, and pancreatic cancers (12–17). In contrast, upregulation of Reelin is observed in retinoblastoma, esophageal carcinoma, high-grade prostate cancer, multiple myeloma, and non-Hodgkin lymphoma (18–22). The alterations in Reelin expression are related to the tumor metastasis and invasion (23, 24). For example, in a human esophageal carcinoma cell line, RELN was related to TGF-β, which was related to metastasis, and knockdown of RELN increased the rate of cell migration (25). However, the role of Reelin in OSCC is far from known.

Although the studies related to Reelin mainly focuses on brain development and tumor progression, some studies have proved that Reelin is also linked to immune function. For example, some studies pointed out that interleukin (IL)-1β mRNA was highly expressed in mice with RELN mutation by stimulating with lipopolysaccharide (26, 27). Decreased Reelin was associated with the alterations in serotonin transporter (SERT) clustering in blood lymphocytes, which may be essential in some cardiovascular or immune system alterations (28). Thus, correlation between Reelin and immune balance in OSCC remains elucidated.

Therefore, in the present study, we focused on the expression pattern of Reelin in OSCC, including tumor cells (Reelin^TC^), cancer**-**associated fibroblasts (Reelin^CAF^), and tumor-infiltrating lymphocytes (Reelin^TIL^). Furthermore, we evaluated the correlation between Reelin and clinicopathological characteristics, its prognostic significance, and immune cell infiltrations.

## Materials and Methods

### Patients and Samples

All methods used for this study were approved by the Ethics Committee of Nanjing Stomatology Hospital [2019NL-009(KS)]. The study was carried out in accordance with the Declaration of Helsinki. Written informed consent was obtained from all the patients. Seventy-five OSCC patients were recruited in this study. All the 75 cases included 14 cases of cheek cancer, 30 cases of tongue cancer, 16 cases of gingival cancer, and 15 cases of other types of oral cancer. The inclusion and exclusion criteria of patients were the same as those of our previous studies (29). Specifically, the patients must have primary OSCC without anti-cancer therapies including chemotherapy and/or radiotherapy before surgery, and no distant metastasis prior to surgery, and these patients underwent curative resection between 2013 and 2016 at Nanjing Stomatology Hospital. Lactating individuals, pregnant women, and patients who were diagnosed with other malignant diseases or certain autoimmune diseases were excluded. These patients with primary tumor were diagnosed by hematoxylin and eosin staining by two experienced pathologists. All 75 patients were followed-up until April 2020. Paraffin-embedded OSCC tissue slices were obtained from the pathology department and used for IHC study. Thirty-nine blood samples from OSCC patients were obtained for flow cytometry assay before any related treatments.

### Immunohistochemistry and Quantification

The protocol of IHC of formalin-fixed paraffin-embedded sections and scoring details of IHC was performed as previously described (29, 30). Specifically, IHC was performed on 3-µm formalin-fixed paraffin-embedded sections using anti-Reelin (monoclonal, 1:100 dilution; MAB5366; Abcam) and anti-Ki-67 (monoclonal, 1:200 dilution; ab16667; Abcam). Slides were deparaffinized with xylene and rehydrated in an ethanol series. Antigen retrieval was performed with 10 mmol/L citric acid (pH 6.0) in a pressure cooker. Then, endogenous peroxidase activity was blocked with a 3% hydrogen peroxide solution. After washing in phosphate-buffered saline (PBS; pH 7.4) three times, slides were incubated with primary antibodies against Reelin (MAB5366; Abcam) at 4°C overnight. Polink-2 plus HRP Detection Kit was used as the secondary antibody at 37°C for 40 min. Finally, slides were developed in diaminobenzidine (DAB). The primary antibody was replaced by PBS as negative control. The IHC staining results of Reelin and Ki-67 were independently and double blindly evaluated by two senior pathologists who did not know the patients’ data, and the average values were calculated for further analysis. IHC staining was scored according to the percentage of positive cells and staining intensity. The percentage of stained cells was defined as 0 = 0–5%; 1 = 6%–25%; 2 = 26%–50%; 3 = 51%–75%; and 4 = 75–100%. Staining intensity was defined as follows: 0 = negative staining; 1 = weak staining; 2 = moderate staining; and 3 = strong staining (29, 30). The IHC score was calculated by multiplying the grade of the staining intensity by that of the staining percentage. High and low expressions of Reelin were defined by the median of IHC scores.

### Primary CAFs Cultivation

CAFs used in this study were isolated from fresh 13 OSCC tumor tissues immediately after surgical resection. Details of this protocol were the same as previously described (29).

### Immunofluorescence

Immunofluorescence performed on primary CAFs was carried out as previously described (29): primary antibodies, α-SMA (1:200, Abcam, England), FSP-1 (1:200, Abcam, England), and PDGFR-β (1:200, Abcam, England); secondary antibodies, Alexa Fluor 647-conjugated (1:100, Abcam, England), Alexa Fluor 555-conjugated (1:200, Abcam, England), and Alexa Fluor 488-conjugated (1:400, Abcam, England).

### mRNA Isolation and Quantitative PCR

Thirteen cultured primary CAFs were classified as WPOI = 1–3 or WPOI = 4–5. The details of extracting total mRNA and conducting quantitative PCR (q-PCR) were performed as previously described (29). Comparative 2^−ΔCt^ or 2^−ΔΔCt^ method was used to quantify the relative mRNA expression as indicated.

### Flow Cytometry Assay

For the cell subtypes of peripheral blood mononuclear cell (PBMC) analysis, details of flow cytometry were the same as previously described (31).

### Analysis of GEO, TCGA, TISIDB, and TIMER Databases

The differential expression of Reelin between CAFs and TCs was performed by Gene Expression Omnibus (GEO, https://www.ncbi.nlm.nih.gov/geo/) database analysis in specific tumors. The Cancer Genome Atlas (TCGA, https://cancergenome.nih.gov/abouttcga/overview) database was used to further validate survival implication of Reelin. Tumor Immune Estimation Resource (TIMER, https://cistrome.shinyapps.io/timer/) was used to learn the association between Reelin mRNA expression and the expression of particular immune infiltrating cell subset markers according to the TIMER’s online instructions. An integrated repository portal for tumor–immune system interactions (TISIDB, http://cis.hku.hk/TISIDB/index.php) was utilized to examine Reelin mRNA and immune system interactions in 28 types of TILs across human cancers.

### Statistical Analysis

SPSS 25.0 software and GraphPad Prism 8.0 were used for statistical analyses and figure process. The results of the experiments were presented as mean ± SEM. Chi-square test, Pearson’s chi-square test, or Fisher’s exact test was used to compare clinicopathological parameters of the patients. Kolmogorov-Smirnov tests were used to learn the relationship between Reelin and Ki-67. The Mann–Whitney *U* test was used to compare the two groups (e.g., Reelin in CAFs with relapse, with relapse *versus* without relapse). Survival analysis including overall survival (OS), disease-specific survival (DSS), and progression-free survival (PFS) were evaluated by Kaplan–Meier survival test. The Cox regression model was used to evaluate the correlations between different prognostic factors in a multivariate analysis. The immune infiltration level was compared with the *t* test. All the analyses were two-sided test and differences were considered statistically significant with *p* < 0.05.

## Results

### The Reelin Levels Were Higher in CAFs Than That in TCs

**Figure 1:**
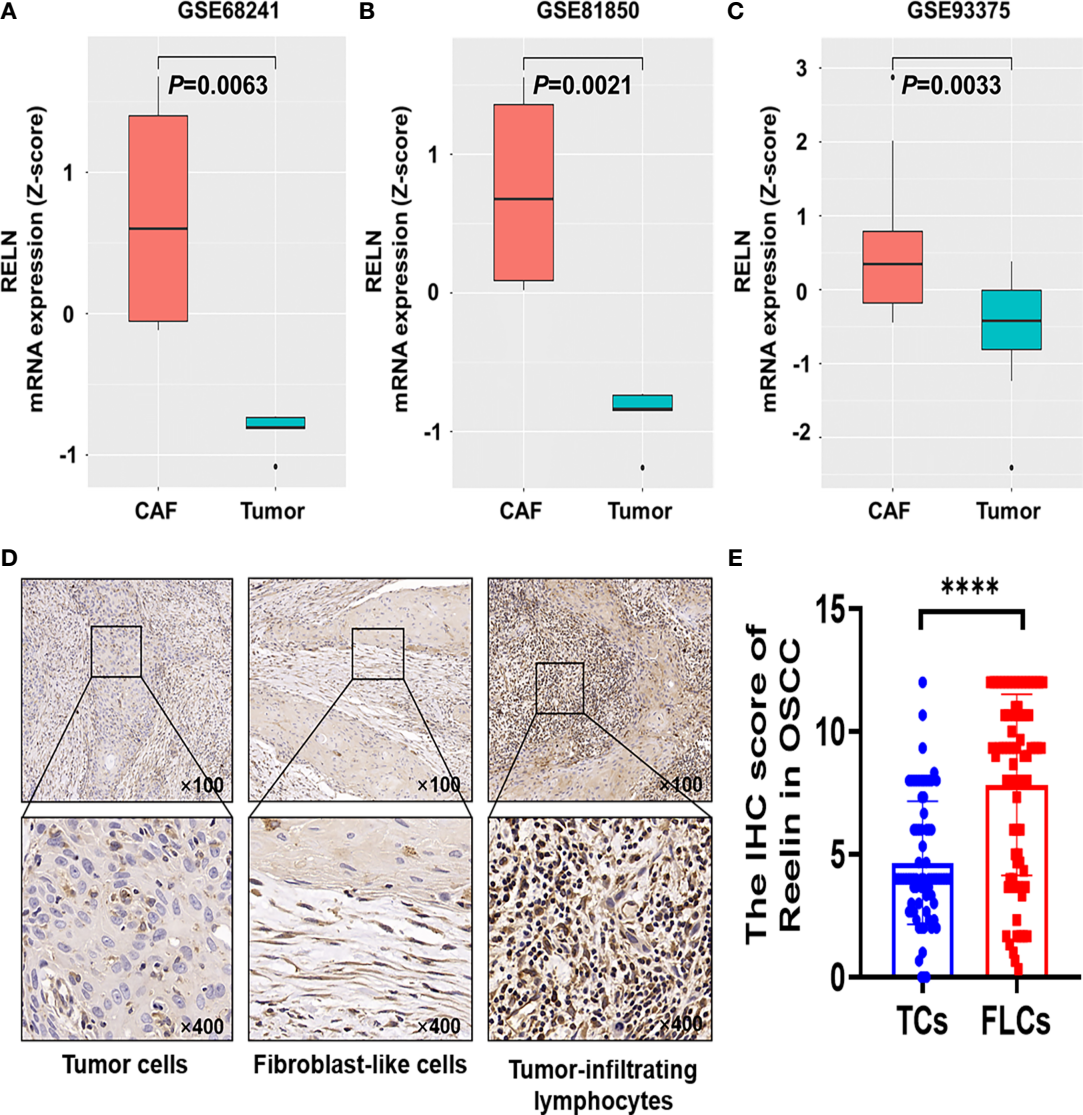
Reelin was upregulated in CAFs compared with TCs derived from OSCC and other several cancers. Enhanced level of RELN^CAF^ compared with RELN^TC^ in breast cancer **(A)**, pancreatic cancer **(B)**, and rectal carcinoma **(C)** using the GEO database. Representative immunohistochemistry staining of Reelin in OSCC, according to TCs, FLCs and TILs **(D)**. The IHC score of Reelin^TC^ and Reelin^FLC^ in OSCC **(E)**. **** denoted that differences were considered statistically significant with p < 0.0001.

Based on the GEO database, we found that Reelin was significantly upregulated in CAFs compared with TCs (*p* = 0.0063, *p* = 0.0021, and *p* = 0.0033; **Figures 1A–C**). Thus, to validate Reelin expression in OSCC, IHC staining was performed to assess the expression of Reelin in different cell types including TCs, FLCs, and TILs. Consistently with the database, the IHC score of Reelin^FLC^ was significantly higher than that of Reelin^TC^ in OSCC (*p* < 0.0001 **Figure 1E**). In addition, representative IHC staining is shown in **Figure 1D**.

### Reelin^CAF^ Positively Correlated With pN stage and WPOI, but Reelin^TC^ Negatively Correlated With pN Stage

**Figure 2:**
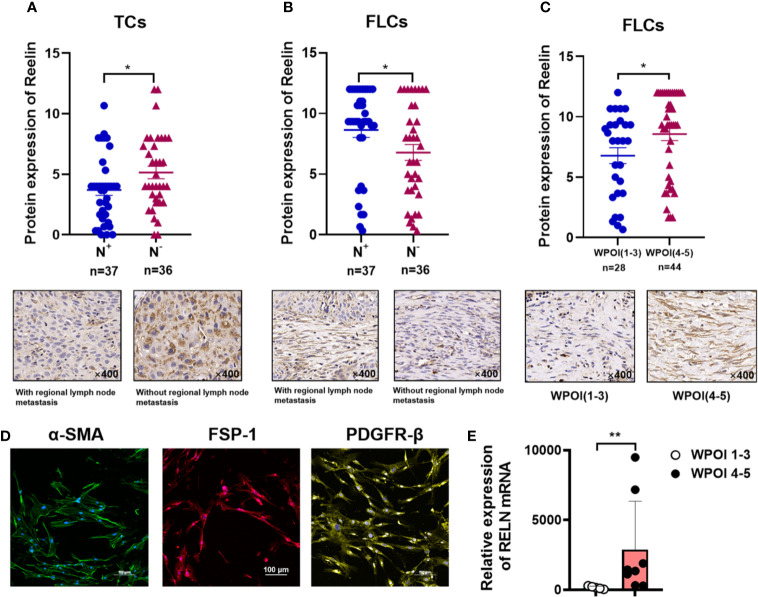
Reelin expression with regional lymph node metastasis in TCs **(A)**, FLCs **(B)**, and WPOI in FLCs **(C)**. Immunofluorescence of isolated primary fibroblasts showed that CAFs express α-SMA, FSP-1, and PDGFR-β **(D)**. Quantitative analysis of RELN mRNA between WPOI (1-3) and WPOI (4-5) revealed significant difference between two groups **(E)**. * and ** denote that differences were considered statistically significant with *p* < 0.05 and *p* < 0.01, respectively.

**Figure 3:**
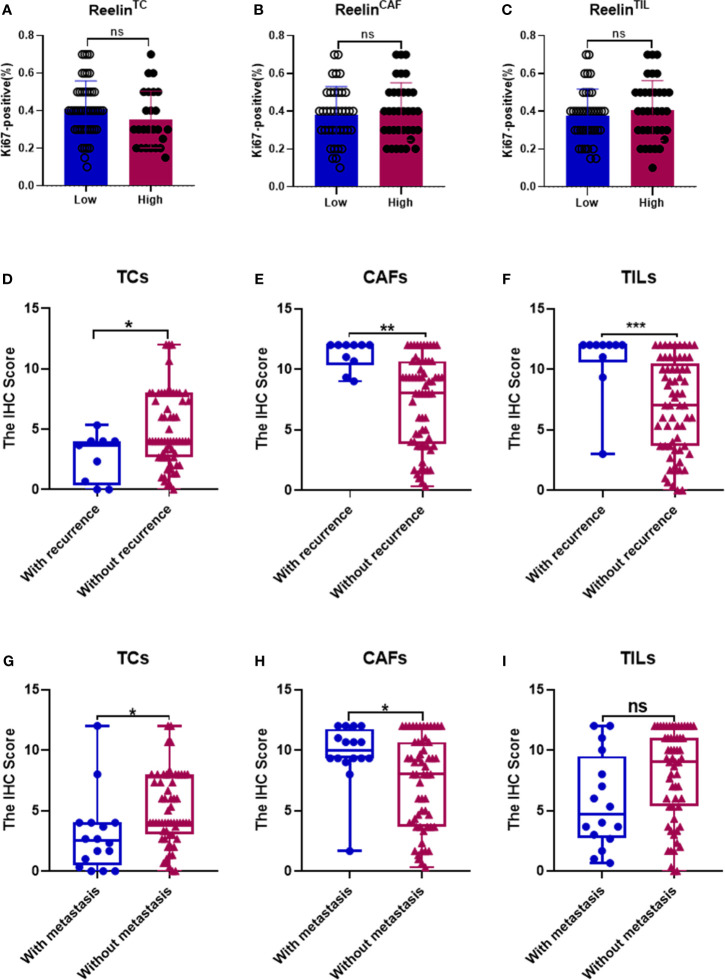
Correlation between Ki-67 in TCs and Reelin in TCs, CAFs, and TILs **(A–C)**. Correlation between Reelin and postoperative recurrence as well as distant metastasis, according to TCs **(D, G)**, CAFs **(E, H)**, and TILs **(F, I)**. *, ** and *** denote that differences were considered statistically significant with *p* < 0.05, *p* < 0.01 and *p* < 0.001 respectively. ns represented no statistical differences.

After confirming the contrary expression pattern of Reelin^TC^ and Reelin^FLC^ in OSCC, we further evaluated the correlation between Reelin and various clinicopathological variables according to different cell types in **Table 1**. The results demonstrated that patients with low-expressed Reelin^TC^ (*p* = 0.041, **Figure 2A**) but high-expressed Reelin^FLC^ (*p* = 0.007, **Figure 2B**) had a high risk of lymph node metastasis. Also, more Reelin^FLC^ was closely linked to poor WPOI (*p* = 0.034, **Figure 2C**). In addition, we continued to study the role of Reelin in primary CAFs from OSCC patients. CAFs were successfully isolated and cultured from fresh OSCC tissues with reliable phenotypic identification using immunofluorescence (**Figure 2D**). We divided CAFs into WPOI (1–3) and WPOI (4–5) groups. Consistently with the IHC results, high-expressed Reelin in primary CAFs had a poor WPOI using qPCR analysis (*p* = 0.0016, **Figure 2E**). However, there were no differences between Reelin and sex, age, smoking habit, T stage, or differentiation (all *p*>0.05). Ki-67 IHC staining also showed that there was no difference between low Reelin levels and high Reelin levels in TCs (*p* = 0.1232, **Figure 3A**), CAFs (*p* = 0.6257 **Figure 3B**), and TILs (*p* = 0.4325, **Figure 3C**).

**Table 1:** Association between Reelin and clinicopathological characteristics in OSCC patients.

Characteristics	TCs					FLCs					TILs				
	n	Low (%)	High (%)	*χ* ^2^	*p*	*n*	Low (%)	High (%)	*χ* ^2^	*p*	*n*	Low (%)	High (%)	*χ* ^2^	*p*
Sex															
Female	29	17 (58.6)	12 (41.4)	0.331	0.565	29	16 (55.2)	13 (44.8)	0.645	0.422	29	17 (58.6)	12 (41.4)	1.197	0.274
Male	46	30 (65.2)	16 (34.8)			46	21 (45.7)	25 (54.3)			46	21 (55.3)	25 (67.6)		
Age															
<60	36	21 (58.3)	15 (41.7)	0.556	0.456	36	15 (41.7)	21 (58.3)	1.628	0.202	36	21 (58.3)	15 (41.7)	1.628	0.202
≥60	39	26 (66.7)	13 (33.3)			39	22 (56.4)	17 (43.6)			39	17 (43.6)	22 (56.4)		
Smoking															
No	33	16 (48.5)	17 (51.5)	2.082	0.149	33	17 (51.5)	16 (48.5)	0.012	0.912	33	17 (51.5)	16 (48.5)	0.194	0.660
Yes	22	15 (68.2)	7 (31.8)			22	11 (50.0)	11 (50.0)			22	10 (45.5)	12 (54.5)		
Not achieved	20					20					20				
T Stage															
1–2	60	38 (63.3)	22 (36.7)		0.760	60	31 (51.7)	29 (48.3)	0.13	0.719	60	29 (48.3)	31 (51.7)	0.745	0.388
3–4	13	9 (69.2)	4 (30.8)			13	6 (46.2)	7 (53.8)			13	8 (61.5)	5 (38.5)		
Not achieved	2					2					2				
N Stage															
No	36	19 (52.8)	17 (47.2)	4.172	0.041*	36	24 (66.7)	12 (33.3)	7.258	0.007*	36	15 (41.7)	21 (58.3)	2.311	0.128
Yes	37	28 (75.7)	9 (24.3)			37	13 (35.1)	24 (64.9)			37	22 (59.5)	15 (40.5)		
Differentiation															
Well	16	7 (43.8)	9 (56.3)	2.942	0.086	16	9 (56.3)	7 (43.8)	0.319	0.572	16	8 (50.0)	8 (50.0)	0.015	0.903
Moderate to poor	58	39 (67.2)	19 (32.8)			58	28 (48.3)	30 (51.7)			58	30 (51.7)	28 (48.3)		
Not achieved	1					1					1				
WPOI															
1–3	28	18 (64.3)	10 (35.7)	0.003	0.955	28	18 (64.3)	10 (35.7)	4.506	0.034*	28	12 (42.9)	16 (57.1)	0.935	0.334
4–5	44	28 (63.6)	16 (36.4)			44	17 (38.6)	27 (61.4)			44	24 (54.5)	20 (45.5)		
Not achieved	3					3					3				

TCs, tumor cells; FLCs, fibroblast-like cells; TILs, tumor-infiltrating lymphocytes; WPOI, worst pattern of invasion; χ^2^, Pearson’s chi-squared test.

*Denotes that differences were considered statistically significant with p < 0.05.

### Upregulated Reelin^CAF^, but Downregulated Reelin^TC^ Correlated With High Risk of Postoperative Relapse, Metastasis, and Poor Clinical Outcome

**Figure 4:**
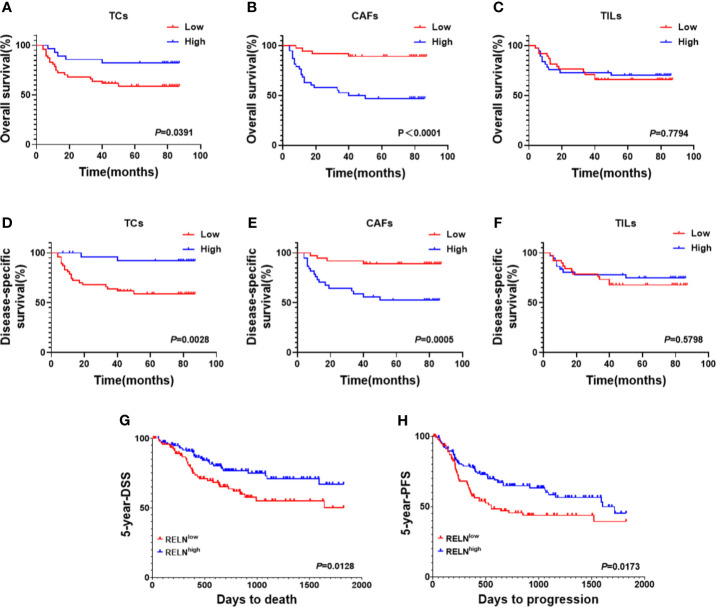
Kaplan–Meier survival curves of OS **(A–C)** and DSS **(D–F)** for OSCC patients according to expression level of Reelin in TCs, CAFs, and TILs. Based on the TCGA database (TCGA.HNSC.sampleMap/HiSeqV2), Kaplan–Meier survival curves of DSS **(G)** and PFS **(H)** for OSCC patients according to expression level of Reelin.

Considering that decreased Reelin^TC^, but increased Reelin^CAF^ was correlated with poor clinicopathological characteristics, we further analyzed the association between Reelin and postoperative relapse as well as distant metastasis according to distinct cell types. The results indicated that patients with postoperative relapse had a low expression of Reelin^TC^ (*p* = 0.0465, **Figure 3D**), but a high expression of Reelin ^CAF^ (*p* = 0.0015, **Figure 3E**). Moreover, patients with low-expressed Reelin^TC^ (*p* = 0.0221, **Figure 3G**) but high-expressed Reelin^CAF^ (*p* = 0.0165 **Figure 3H**) were prone to postoperative metastasis. Strikingly, patients with high Reelin^TIL^ also had a high risk of postoperative relapse (*p* = 0.0007, **Figure 3F**). Reelin in TILs didn't have relationship with metastasis (P = 0.0565, **Figure 3F**). Considering that the role of Reelin in TCs was contrary to that in CAFs in cancer metastasis and relapse, we further evaluated its prognostic value in OS and DSS. Kaplan–Meier curve analysis showed that patients with lower-expressed Reelin^TC^ but higher-expressed Reelin^CAF^ had a shorter OS (*p* = 0.0391, **Figure 4A**; *p*<0.0001, **Figure 4B**) and DSS (*p* = 0.0028, **Figure 4D**; *p* = 0.0005, **Figure 4E**). Reelin in TILs wasn’t linked with OS and DSS (P = 0.7794, **Figure 4C**; P = 0.5798, **Figure 4F**). Consistent with our results, the TCGA database (TCGA.HNSC.sampleMap/HiSeqV2) also showed that patients with high-expressed Reelin^TC^ had a longer DSS (*p* = 0.0128, **Figure 4G**) and PFS (*p* = 0.0173, **Figure 4H**).

### Reelin^CAF^ Was an Independent Prognostic Factor of OS and DSS

Univariate and multivariate analyses suggested that Reelin^CAF^ was a valuable prognostic factor among the clinicopathologic variables examined, including gender, age, smoking, T stage, N stage, differentiation, and WPOI. Note that patients with high-expressed Reelin^CAF^ had a worse OS (*p* = 0.004, **Table 2**) and DSS (*p* = 0.009, **Table 2**). These data clearly showed that Reelin^CAF^ level may further refine the prognostic criteria so as to find OSCC patients who may need extra therapy.

**Table 2:** Cox-regression analyses of overall survival (OS) and disease-specific survival (DSS) in OSCC patients.

Variables	Univariate analysis		*p*	Multivariate analysis		*p*
	Hazard ratio	95% CI	**	Hazard ratio	95% CI	**
**OS**						
Gender						
Male *vs*. Female	1.46	0.625–3.413	0.382			
Age						
<60 *vs*. ≥60	0.992	0.446–20210	0.985			
Smoking						
No *vs*. Yes	1.073	0.408–20820	0.886			
T Stage						
1–2 *vs*. 3–4	1.374	0.513–3.684	0.527			
N Stage						
No *vs*. Yes	9.828	2.919–33.088	<0.001*	7.618	2.242–25.887	0.001*
Differentiation						
Well *vs*. Moderate to poor	2.164	0.645–7.260	0.211			
WPOI						
1–3 *vs*. 4–5	2.954	1.102–7.921	0.031*	2.767	1.025–7.471	0.045*
Reelin^TC^						
Low *vs*. High	2.589	0.966–6.941	0.059			
Reelin^CAF^						
Low *vs*. High	0.148	0.051–0.435	0.001*	0.202	0.068–0.599	0.004*
Reelin^TIL^						
Low *vs*. High	0.922	0.413–2.061	0.844			
						
**DSS**						
Gender						
Male *vs*. Female	1.461	0.590–3.622	0.413			
Age						
<60 *vs*. ≥60	0.902	0.383–2.125	0.814			
Smoking						
No *vs*. Yes	1.024	0.364–2.878	0.964			
T Stage						
1–2 *vs*. 3–4	1.235	0.415–3.673	0.705			
N Stage						
No *vs*. Yes	8.504	2.492–29.016	0.001*	4.776	1.345–16.962	0.016*
Differentiation						
Well *vs*. Moderate to poor	1.852	0.545–6.293	0.323			
WPOI						
1–3 *vs*. 4–5	2.499	0.914–6.831	0.074			
Reelin^TC^						
Low *vs*. High	6.485	1.509–27.866	0.012*	4.122	0.941–18.053	0.06
Reelin^CAF^						
Low *vs*. High	0.173	0.058–0.516	0.002*	0.226	0.074–0.691	0.009*
Reelin^TIL^						
Low *vs*. High	0.811	0.341–1.928	0.636			

CI, confidence interval; Reelin^CAF^, Reelin in cancer**-**associated fibroblasts; Reelin^TC^, Reelin in tumor cells; Reelin^TIL^, Reelin in tumor-infiltrating lymphocytes; WPOI, worst pattern of invasion.

*Denotes that differences were considered statistically significant with p < 0.05.

### Reelin^TIL^ Disrupted the Balance of CD4^+^/CD8^+^ T Cell in Peripheral Blood

**Figure 5:**
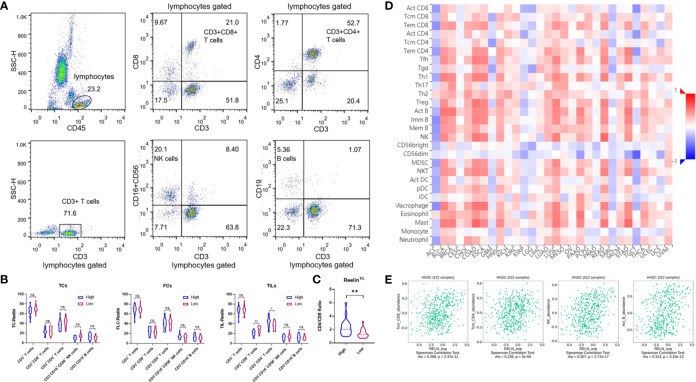
Flow cytometry analysis of lymphocytes, gated based on characteristic light-scatter properties, and single lymphocytes were gated based on forward scatter height *vs*. forward scatter area (FSC-A). The numbers in the quadrants or over line indicate the percentage of cells **(A)**. Correlation of Reelin expression with immune infiltration level in OSCC before surgery, according to TCs, CAFs, and TILs **(B)**. Correlation of CD4/CD8 ratio with Reelin in TILs **(C)**. Relations between expression of RELN and 28 types of TILs across human heterogeneous cancers **(D)**. RELN significantly correlated with CD8^+^ T cells, CD4^+^ T cells, NK cells, and B cells in HNSCC **(E)**. * and ** denote that differences were considered statistically significant with *p* < 0.05 and *p* < 0.01, respectively. ns represented no statistical differences.

Several previous studies have pointed out that Reelin also played a vital role in immune function. Our data showed that patients with high-expressed Reelin^TIL^ had a high risk of postoperative relapse. We further analyzed the correlation between Reelin and lymphocyte subsets from PBMCs of OSCC using flow cytometry. Lymphocytes were gated as shown in **Figure 5A**. The results demonstrated that patients with high-expressed Reelin^TIL^, not Reelin^TC^ or Reelin^CAF^, had a low ratio of CD3^+^CD8^+^ T cells (*p* = 0.0085, **Figure 5B**) and a high ratio of CD3^+^CD4^+^ T cells (*p* = 0.0118, **Figure 5B**). A study reported that elevated CD4/CD8 ratio was related to poor survival in head and neck squamous cancer (32). Therefore, we evaluated the CD4/CD8 ratio between low and high expression of Reelin^TIL^. The result showed that more Reelin^TIL^ had a higher ratio of CD4/CD8 (*p* = 0.0018, **Figure 5C**). Besides, our data showed no relationship between Reelin and B cells as well as NK cells from peripheral blood.

### Reelin^TIL^ Promoted Multitype Immune Cells Infiltration in the Tissue Microenvironment

We furthermore evaluated the relationship between RELN and tumor immune microenvironment. We found correlations of RELN with 28 types of TILs across human heterogeneous cancers according to the TISIDB database (**Figure 5D**). In HNSCC, RELN positively correlated with abundance of central memory CD8 T cells (Tcm_CD8 T cells; rho = 0.288, *p* < 0.001, **Figure 5E**), central memory CD4 T cells (Tcm_CD4 T cells; rho = 0.236, *p* < 0.001, **Figure 5E**), natural killer cells (NK cells; rho = 0.357, *p* < 0.001, **Figure 5E**), and activated B cells (B cells; rho = 0.313, *p* < 0.001, **Figure 5E**). Meanwhile, the Spearman’s correlation test was further used to examine the correlation among RELN and immune cell infiltration of HNSCC according to the TIMER database (**Table 3**). Elevated RELN was significantly associated with T-cell, B-cell, monocyte, macrophage, and dendritic cell infiltration (all *p* < 0.05), leading to a multitype immune cell infiltration in the tissue microenvironment.

**Table 3:** Correlation analysis between RELN and immune cell infiltrations in HNSCC samples using TIMER.

Description	Gene markers	RELN			
		None		Purity	
		Cor	*p*	Cor	*p*
CD8+ T cell	CD8A	0.185	***	0.134	**
	CD8B	0.162	***	0.114	*
T cell (general)	CD3D	0.182	****	0.129	*
	CD3E	0.264	****	0.217	****
	CD2	0.258	****	0.211	****
B cell	CD19	0.23	****	0.186	****
	CD79A	0.236	****	0.196	****
Monocyte	CD86	0.345	****	0.307	****
	CSF1R	0.397	****	0.358	****
TAM	CCL2	0.413	****	0.383	****
	CD68	0.279	****	0.245	****
	IL10	0.424	****	0.395	****
M1 macrophage	NOS2	0.166	***	0.185	****
	IRF5	0.028	0.525	0.025	0.578
	PTGS2	0.169	***	0.213	****
M2 macrophage	CD163	0.348	****	0.308	****
	VSIG4	0.261	****	0.219	****
	MS4A4A	0.3	****	0.252	****
Neutrophils	CEACAM8	−0.017	0.703	−0.026	0.564
	ITGAM	0.27	****	0.24	****
	CCR7	0.383	****	0.342	****
Natural killer cell	KIR2DL1	0.067	0.127	0.061	0.18
	KIR2DL3	0.055	0.209	0.038	0.406
	KIR2DL4	0.058	0.186	0.044	0.334
	KIR3DL1	0.164	***	0.144	**
	KIR3DL2	0.167	***	0.137	**
	KIR3DL3	−0.027	0.544	−0.04	0.373
	KIR2DS4	0.035	0.423	0.007	0.878
Dendritic cell	HLA-DPB1	0.28	****	0.232	****
	HLA-DQB1	0.217	****	0.182	****
	HLA-DRA	0.285	****	0.236	****
	HLA-DPA1	0.299	****	0.252	****
	CD1C	0.382	****	0.35	****
	NRP1	0.458	****	0.429	****
	ITGAX	0.272	****	0.232	****
Th1	TBX21	0.191	****	0.15	***
	STAT4	0.287	****	0.25	****
	STAT1	0.208	****	0.176	****
	IFNG	0.034	0.436	−0.01	0.822
	TNF	0.2	****	0.193	****
Th2	GATA3	0.307	****	0.273	****
	STAT6	0.249	****	0.27	****
	STAT5A	0.258	****	0.24	****
	IL13	0.204	****	0.186	****
Tfh	BCL6	0.097	0.0271	0.126	**
Th17	STAT3	0.312	****	0.317	****
	IL17A	0.195	****	0.162	***
Treg	FOXP3	0.391	****	0.356	****
	CCR8	0.49	****	0.468	****
	STAT5B	0.395	****	0.387	****
	TGFB1	0.251	****	0.246	****
T cell exhaustion	PDCD1	0.165	***	0.114	*
	CTLA4	0.247	****	0.203	****
	LAG3	0.107	*	0.068	0.133
	HAVCR2	0.279	****	0.238	****
	GZMB	0.087	*	0.045	0.315

TIMER, Tumor IMmune Estimation Resource; TAM, tumor-associated macrophage; Th, T helper cell; Tfh, Follicular helper T cell; Treg, regulatory T cell; Cor, R value of Spearman’s correlation; None, correlation without adjustment; Purity, correlation adjusted by purity.

*, **, ***, and **** denote that differences were considered statistically significant with p < 0.05, p < 0.01, p < 0.001, and p < 0.0001, respectively.

## Discussion

Reelin has a great impact on carcinogenesis and tumor progression. However, the expression of Reelin in different cancers was different. For example, Reelin is decreased in hepatocellular carcinoma, but increased in esophageal carcinoma (13, 22). Based on the GEO database, we found that Reelin was significantly upregulated in CAFs compared with TCs in breast cancer, pancreatic cancer, and rectal carcinoma. However, the expression of Reelin in OSCC had not yet been investigated. In this study, we examined the expression pattern of Reelin in OSCC for the first time. The result also demonstrated that Reelin levels were higher in CAFs compared with TCs in OSCC. Besides, TIL-derived Reelin was expressed distinctively according to different OSCC patients. Therefore, we concluded that Reelin might have a versatile function in different cell types during the development of OSCC *via* governing tumor cell and stroma microenvironment.

According to the significant role of Reelin in tumor progression, some studies showed that upregulated Reelin was found to be correlated with proliferation of cancer cells in multiple myeloma (33). However, stimulating Reelin signaling showed that the proliferation of tumor cells in glioblastoma decreased significantly (15). Moreover, in breast cancer, a previous study showed that enhanced Reelin in MDA-MB231 cells suppressed invasiveness of cancer cells *in vitro* (17). In OSCC, to investigate the association between Reelin and tumor proliferation, we analyzed Ki-67 among high- and low-expressed groups of Reelin. The result showed that there was no statistical relationship between low and high Reelin levels. However, patients with high-expressed Reelin^CAF^ were predicted to have a poor WPOI. Notably, low-expressed Reelin^TC^, but high-expressed Reelin^CAF^ was related to the lymph node metastasis, which indicated that Reelin has an opposite function in CAFs and TCs during the development of OSCC.

Besides being related to clinicopathological parameters, some previous studies also determined that Reelin is also related to the prognosis of patients. For example, in hepatocellular carcinoma (HCC), relapse was more likely to happen when the expression of Reelin in tumor cells was high (13). In addition, comparing metastatic colorectal diseases with primary tumors, the expression of Reelin increased significantly in metastatic tumors (23). These data indicated that Reelin might play a vital role in local relapse and distant metastasis. In our survival analysis, the result showed that patients with higher-expressed Reelin^CAF^ had a greater risk of relapse and distant metastasis. Instead, patients with higher-expressed Reelin^TC^ had a lower risk of relapse and distant metastasis. Strikingly, patients with high-expressed Reelin^TIL^ also had a high risk of relapse. This finding further suggested that Reelin^CAF^ and Reelin^TC^ play a diverse role in the prognosis of OSCC patients. Also, Reelin^TIL^ might be a potential indicator with an increased risk of OSCC relapse.

Moreover, as a previous study showed, high-expressed Reelin was negatively associated with PFS and OS in multiple myeloma (34). However, low-expressed Reelin significantly correlated with poor clinical outcome in breast cancer (17). Also, in glioblastoma, the expression of Reelin was positively related to survival (15). Just as we predicted, the result showed that higher-expressed Reelin^CAF^, but lower-expressed Reelin^TC^ is correlated with shorter OS and DSS in this study. Meanwhile, Reelin^CAF^ was an independent prognostic factor of OS and DSS for OSCC patients.

Furthermore, Reelin-related studies had focused on not only the tumor progression but also the immune function. For example, alterations in serotonin transporter (SERT) clustering in blood lymphocytes associated with a decrease in Reelin expression may be operative in some cardiovascular or immune system alterations (28). Our data showed that patients with high-expressed Reelin^TIL^ had a high risk of postoperative relapse. We identified the relationship between the expression of Reelin and lymphocyte subsets from PBMCs of OSCC. The result showed that patients with high Reelin^TIL^, but not Reelin^TC^ and Reelin^CAF^ had poor cytotoxicity of CD8^+^ T cells and higher ratio of CD4/CD8 in peripheral blood, which might result in relapse of OSCC patients. However, the database showed that Reelin was positively associated with tissue-resident B cells and NK cells in the tumor microenvironment, suggesting that Reelin regulates immune response depending on the tissue or blood microenvironment.

In conclusion, we identified that increased Reelin^CAF^ had a higher risk for metastasis and relapse. Therefore, Reelin^CAF^ can be used as a potential biomarker in diagnosis and treatment of OSCC patients. Moreover, Reelin could regulate the balance of CD4^+^/CD8^+^ T cells in peripheral blood, which indicated that Reelin might affect the prognosis of OSCC patients. However, it is positively correlated with tissue-resident B cells and NK cells, suggesting that Reelin regulates immune response depending on the tissue or blood microenvironment. Altogether, the study mainly explored the prognostic value of Reelin and the role in immune imbalance in OSCC. However, the molecular function and regulation pathway of Reelin in tumorigenesis of OSCC remained unexplored. In addition, the molecular mechanism of Reelin on immunomodulatory effects was still quite unclear. Future research is needed to unravel the molecular function and regulation pathway of Reelin in tumorigenesis of OSCC. Moreover, the molecular mechanism of Reelin on immunomodulatory effects need to be further explored.

## Data Availability Statement

The original contributions presented in the study are included in the article/supplementary material. Further inquiries can be directed to the corresponding authors.

## Ethics Statement

All the patients have signed the written informed consent before the study. And the present study was approved by the Research Ethics Committee of Nanjing Stomatological Hospital.

## Author Contributions

All authors contributed to the article and approved the submitted version.

## Funding

This work was supported by the National Natural Science Foundation of China (Grant Nos. 81902754, 81772880, 82002865, and 81702680), the Natural Science Foundation of Jiangsu Province (No. BK20190304), the Fundamental Research Funds for the Central Universities (No. 021014380161), the China Postdoctoral Science Foundation (No. 2019M651789), and the Nanjing Medical Science and Technology Development Foundation, Nanjing Department of Health (Nos. YKK18123 and YKK19091).

## Conflict of Interest

The authors declare that the research was conducted in the absence of any commercial or financial relationships that could be construed as a potential conflict of interest.

## Publisher’s Note

All claims expressed in this article are solely those of the authors and do not necessarily represent those of their affiliated organizations, or those of the publisher, the editors and the reviewers. Any product that may be evaluated in this article, or claim that may be made by its manufacturer, is not guaranteed or endorsed by the publisher.
